# Active intrinsic conductances in recurrent networks allow for long-lasting transients and sustained activity with realistic firing rates as well as robust plasticity

**DOI:** 10.1007/s10827-021-00797-2

**Published:** 2021-10-03

**Authors:** Tuba Aksoy, Harel Z. Shouval

**Affiliations:** 1 Department of Neurobiology and Anatomy, The University of Texas, Medical School, Houston, TX, USA; 2 Department of Experimental Radiation Oncology, The University of Texas MD Anderson Cancer, Center, Houston, TX, USA; 3 MD Anderson and UTH Graduate School, The University of Texas, Houston, TX, USA

**Keywords:** Persitant activity, Synaptic plasticity, Active conductances, Interval timing

## Abstract

Recurrent neural networks of spiking neurons can exhibit long lasting and even persistent activity. Such networks are often not robust and exhibit spike and firing rate statistics that are inconsistent with experimental observations. In order to overcome this problem most previous models had to assume that recurrent connections are dominated by slower NMDA type excitatory receptors. Usually, the single neurons within these networks are very simple leaky integrate and fire neurons or other low dimensional model neurons. However real neurons are much more complex, and exhibit a plethora of active conductances which are recruited both at the sub and supra threshold regimes. Here we show that by including a small number of additional active conductances we can produce recurrent networks that are both more robust and exhibit firing-rate statistics that are more consistent with experimental results. We show that this holds both for bi-stable recurrent networks, which are thought to underlie working memory and for slowly decaying networks which might underlie the estimation of interval timing. We also show that by including these conductances, such networks can be trained to using a simple learning rule to predict temporal intervals that are an order of magnitude larger than those that can be trained in networks of leaky integrate and fire neurons.

## Introduction

**Fig. 1:**
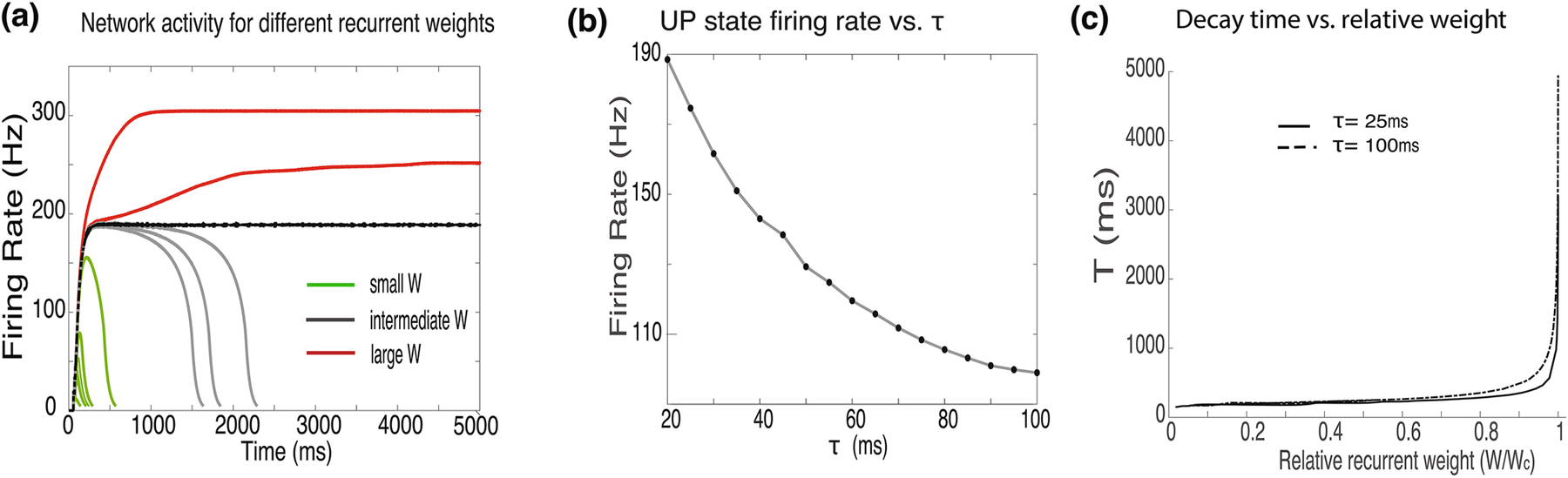
Network behavior for recurrently connected leaky integrate and firing neurons **(a)** The mean firing rate of the network for increasing synaptic weights. The peak value of the firing rate increases for stronger couplings (green lines), and the network decays at a slower rate. At some critical value of recurrent weights, the network becomes bi-stable (black line) as the weights increase further (red lines) the firing rate of the ‘UP’ state increases. The mean firing rate is averaged over all neurons in the network and convolves with an exponential smoothing kernel as explained in the methods section. **(b)** Firing rate of the UP state just above the critical weight, for different synaptic time constants from 20ms to 100ms. **(c)** Decay time (T) increases exponentially for gradually increased synaptic weights (W). The shape of the curve depends on the synaptic time constant. Curves for 100ms synaptic time constant (dashed line) reduces the steepness of the curve slightly compared to 25ms time constant (solid line). The value of W is normalized by Wc; the critical value of the weight parameter at which the network becomes bi-stable

Neurons in the Brain exhibit long-lasting activity that outlasts the typical intrinsic time constants of single neurons by orders of magnitude (Fuster & Alexander, 1971; Goldman-Rakic, 1995). In some experimental settings, recorded neurons also exhibit long-lasting activity that terminates at intervals with a behavioral significance such as the expected timing of reward (Huertas et al., 2015; Shuler & Bear, 2006). Such experimentally observed behaviors can be accounted for by networks of interacting neurons, and reverberations within these networks can account for the long-lasting time constant of neuronal activity. Such patterns of behaviorally relevant neural dynamics can be learned from examples in experimental settings. Various models have been proposed over the years to demonstrate how such recurrent networks can account for long lasting activity (Compte et al., 2000)(Renart et al., 2004), and for learning temporal intervals (Gavornik & Shouval, 2011; Gavornik et al., 2009). Working memory models have often relied on synapses with slow time constants such as NMDA receptors (Wang, 1999). Such slow synapses were assumed because networks with faster, AMPA like synapses typically exhibit very high firing rates in the self-sustaining persistent activity state (Gavornik & Shouval, 2011; Wang, 1999), and these activity levels are much higher than those observed experimentally. If the network activity is not self-sustained, but receives external input it can be bi-stable and sustain realistic firing rate statistics in the active state even with fast time constants (Renart et al., 2006). There is some evidence that there is a high concentration of NMDA receptors in prefrontal cortex, where many experimental results of persistent activity have originated (Wang et al., 2013). However, even if there is a high concentration of NMDA receptors, it still needs to be shown that these receptors, and not the faster AMPA receptors are the ones that are modified in order to generate these plastic recurrent networks. Similarly, in networks that learn to predict interval timing, slow synaptic conductances have been used as well (Gavornik & Shouval, 2011; Gavornik et al., 2009), in order to avoid unrealistically high firing rates. Additionally, networks with fast, AMPA-type, receptors with realistic variability are hard in practice to train in order to generate temporal intervals that last for more than a few hundred milliseconds. These prior observations and the impact of AMPA-type receptors on network dynamics are explained in more detail below and in figure 1.

Although recurrent networks are the most prominent theory for long-lasting neural activity, an alternative theory with experimental support is that positive activity feedback loops of intrinsic conductances within single cells are able to generate persistent activity (Egorov et al., 2002; Fransén et al., 2006), and such mechanisms can also be generalized to neurons that can learn to predict interval timing. The primary experimental support for such active intrinsic conductances, and their contribution to persistent activity arises from Entorhinal slices, although similar channels are shown to exist in other regions including thalamus (O’Malley et al., 2020). Currently, most evidence that intrinsic conductances play a role in persistent activity arises from in vitro studies.

In this paper, we set up to show that a recurrent network of neurons with active intrinsic channels(Fransén et al., 2006; Tegnér et al., 2002), and with fast synapses, is able to generate persistent activity with low firing rates, and to robustly learn temporal intervals that last more than 10 seconds. In a sense this is a hybrid of the two previous approaches, the positive feedback loop observed in single cells is embedded within each neuron of a network model. Single cells within this network are unable to generate sufficient persistent activity alone, but the intrinsic mechanism contributes to long-lasting activity in combination with the recurrent connections. In such a network, the plasticity that generates these ensembles with long-lasting activity is synaptic plasticity rather than plasticity of the intrinsic channels themselves. In this model, the intrinsic activity feedback loop, acts as a conditional slow time constant; this mechanism is typically turned off at rest, but gets activated by sufficient feedforward input or recurrent network activity. With this hybrid model, networks with fast synapses are able to generate persistent activity while exhibiting biologically plausible firing rates. Also, the intrinsic mechanism allows recurrent networks to be trained robustly to predict interval timing over larger temporal intervals, while exhibiting biologically observed firing rates. The active intrinsic conductnaces generate a conditional slow time constant, which is turned on only when the neuronal activity is sufficiently high. This conditional slow time constant allows the network to have a fast on rate for these states together with persistent or very slowly decaying activity. In contrast, in network models with slow synapses, the convergence to the persistent state is also slowed down when synaptic time constants are long.

## 2. Network dynamics

### 2.1 Dynamics of simple recurrent networks with leaky integrate and fire neurons

Here we display the dynamics of simple networks composed of excitatory leaky integrate and fire (LIF) neurons with no additional intrinsic channels. We use this model to illustrate some of the problems such models encounter in accounting for the experimentally observed results when the synaptic conductances are fast. Such observations have promoted previous models to be based on slow synaptic conductances (Wang, 1999).

Network dynamics, in this model, are determined by the strength of the recurrent connections within the network. Figure 1a, shows the dynamics of a network with fast membrane and synaptic dynamics (τ_m_ = 20ms,τ_s_ = 25ms). Changes in the magnitude of recurrent weights affects the peak of the network activity, the plateau firing level and the duration of the delayed activity (Fig. 1a). For small synaptic weights, the network’s activity decays quickly (green lines), almost indistinguishably from the dynamics of a single cell. As synaptic weights increase (gray lines), the networks dynamics slows down significantly exhibiting a low transient that eventually decays. Such dynamics can be used to represent learned interval times (Gavornik & Shouval, 2011; Gavornik et al., 2009). Peak network activity rates in such a case are very high (Fig. 1a,b), more than 150 Hz. Such high firing rates are inconsistent with experimental findings. As the recurrent weight increase further, firing rate form a plateau (Fig. 1a, Black and red lines). At these weights, the network is bi-stable with two possible stable states; a zero or low firing-rate state called the DOWN state, and a state with rapid firing called the UP state. For these parameters, all persistent activity states have firing rates that exceed 180 Hz. Increased synaptic weights result in higher firing rates (Fig 1a, red lines). The black curve in figure 1a depicts network dynamics for a weight (Wc) which is just above criticality, this UP state has the minimal firing rate possible for these parameters. Note that we have simulated networks with such high firing rates, not because these results are similar to experimental observations, but precisely in order to show that under these assumptions networks do not replicate experimental results.

The firing rates of the UP state depend on the synaptic time constant τ_s_. In figure 1b we show how the firing rate of the UP state depends on τ_s_ in the range of 20–100ms (Fig. 1b). For all synaptic time constant values, the network exhibited the same type of qualitative behavior as the network with fast (τ_s_ = 25ms) synaptic dynamics in figure 1A. However, as τ_s_ increased, the critical bi-stable firing rate monotonically decreased from 188Hz at τ_s_ = 20ms to 100Hz at τ_s_ = 100ms (Fig. 1b). Even the firing rates for a slow synaptic time constant of 100ms are high compared to the activity levels typically observed in brain circuits (Goldman-Rakic, 1995). We have previously obtained similar results analytically with a mean-field theory (Gavornik & Shouval, 2011).

We have been able to tune the weights of the model in order to generate transient activity that lasts for a few seconds (Fig 1a,c). However, with a finite resolution of synaptic efficacies and with neuronal noise, attaining such large durations is not practical. We have explored this slow transient regime, by gradually increasing the synaptic efficacies, and for each efficacy level noting the time it takes the network to decay. We have defined the decay time (T) as the time it takes the network to return to a firing rate of 5Hz. In figure 1D we show how the network’s decay time depends on the synaptic efficacy, for two synaptic time constants τ_s_ = 25ms (solid line), and τ_s_ = 100ms (dashed line). The X axis is the weight divided by the critical weight for obtaining bi-stability. For both time constants these curves start very flat, and as they approach the critical weight value, they become very steep, however the curve for the 100ms time constant is less steep. These steep curves imply that very small changes in synaptic weights result in large changes in the network decay times, and small fluctuations can even cause the network to become bi-stable. Using these deterministic spiking networks, with no added noise, we were not able to produce delayed activity that last longer than 3000ms for τ_s_ = 25ms, and to 5000ms for τ_s_ = 100ms, and this is despite having nearly infinite resolution in setting synaptic efficacies. With minimal limits on the resolution of synaptic efficacies and with minimal noise it is extremely hard to code for durations longer than 900ms and 1600ms decay times, for these different time constants respectively. These results are consistent with our earlier studies that maximum temporal representations were limited to 1–2 seconds (Gavornik and Shouval, 2011).

In previous models a mix of AMPA receptors with fast time constants and NMDA receptors with slow time constants have been used (Compte et al., 2000; Tegnér et al., 2002; Wang, 1999). Our observation that slow synaptic time constants are necessary to obtain experimentally realistic firing rates is equivalent to the previous observations that a high NMDA to AMPA ratio is necessary for obtaining realistic firing rates in the UP state. Simply adding recurrently connected inhibitory neurons does not generate a self-sustaining bi-stable network with realistic firing rates, and external currents must be added to produce bi-stable networks where the UP state has low firing rates (Renart et al., 2006).

### 2.2 Single cell model with positive-feedback active currents

Besides the models that account for persistent activity by reverberation in networks with recurrent connections, there is also experimental evidence and theoretical studies (Egorov et al., 2002; Fransén et al., 2006), (O’Malley et al., 2020; Shouval & Gavornik, 2011) that show persistent activity and slowly decaying activity can be accounted for by a feedback loop of different intrinsic conductances. Specifically, the experimental data suggests models in which calcium activated non-selective cation channels together with voltage dependent calcium channels create an intracellular positive feedback loop that keeps the cell firing for an extended time period.

**Fig. 2:**
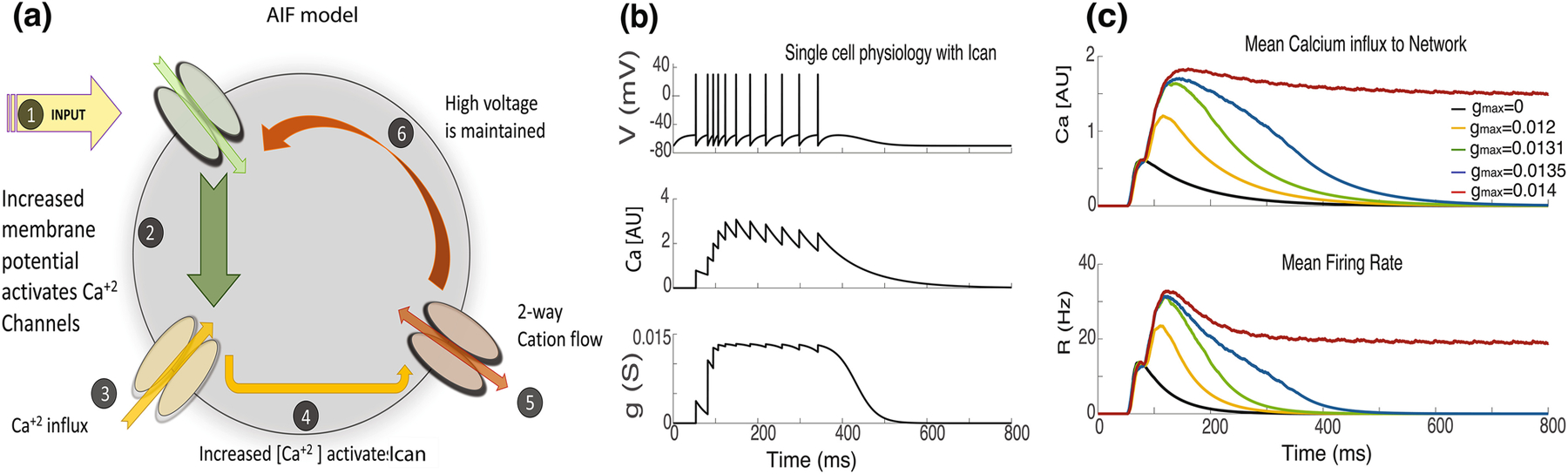
Dynamics of single AIF neuron. **(a)** Each neuron is built up with an excitatory input channel, a high voltage activated (HVA) calcium channel, a non-selective cation conductance, and a leakage conductance. The input elevates the membrane voltage and initiates firing of the neuron. With each spike calcium current flows into the cell. Increased levels of intracellular calcium activate the I_CAN_ conductance. The inward cation current maintains the high levels of membrane voltage. This feedback loop maintains the persistent neural firing. **(b)** The dynamics of single neuron with I_CAN_ currents following 100ms of external stimuli. The subplots show the membrane voltage, intracellular calcium concentration and I_CAN_ conductance. **(c)** The network is simulated for different g_max_ values, the traces are coded with matched colors for corresponding [Ca] and firing rate

The basic conductance based LIF model describes the membrane potential within a single compartment with a leakage and an input current. In this section we add to the single cell model active intrinsic conductances that control the subthreshold depolarization, but spikes in this model are still initiated by threshold crossing and not by voltage dependent conductances as in the Hodgkin-Huxley formulation. Each neuron consists of excitatory input channel, high voltage activated (HVA) calcium channel, non-selective cation conductance (I_CAN_), and leakage conductance (Fig. 2a). We call this type of neuron an active integrate and fire neuron (AIF) (See Methods, Section 3 for details). The neuron receives synaptic input from the external population and generates action potentials, as the membrane voltage exceeds the threshold value. With each spike, the HVA open up briefly and allows calcium flow into the cell. These currents increase intracellular calcium levels to activate calcium dependent non-selective cation channels (I_CAN_) (Fig 2a).

The dynamics of a single neuron following a transient input of 100ms are shown in figure 2b. The initial external activation of the cells causes action potentials which generate activation of calcium currents through HVA channels (Fig 2b, center), increased calcium opens the I_CAN_ channels (Fig 2b bottom) which causes sufficient cellular depolarization to generate additional action potentials. This positive feedback loop generates sustained firing. This intracellular feedback loop maintains the firing of the neuron, but at these parameters, the compensation of this loop is slightly less than the leak current, so that cells activity slowly dies down. The effect of I_CAN_ channels on the cellular dynamics depends on its maximum conductance, denoted as g_max_ (Fig. 2c). As g_max_ is increased, the duration of sustained activity is increased. For larger values of g_max_ the single cell becomes bi-stable (Fig. 2c, red). Here we propose to study the impact of including these active conductances in single cells within a network. We will add them with maximal conductances that are subthreshold for single neuron bi-stability. We hypothesize that including these will add a conditional very-slow time constant to the neurons which may both produce network bi-stability at much lower firing rates as well as increase the range of transient activities to encode temporal intervals.

### 2.3. Network dynamics with AIF model

In this section we demonstrate the effects of using AIF neurons within a recurrent network. The network conserves the same input parameters and network structures of the network of LIF neurons. See the methods section for details.

**Fig. 3:**
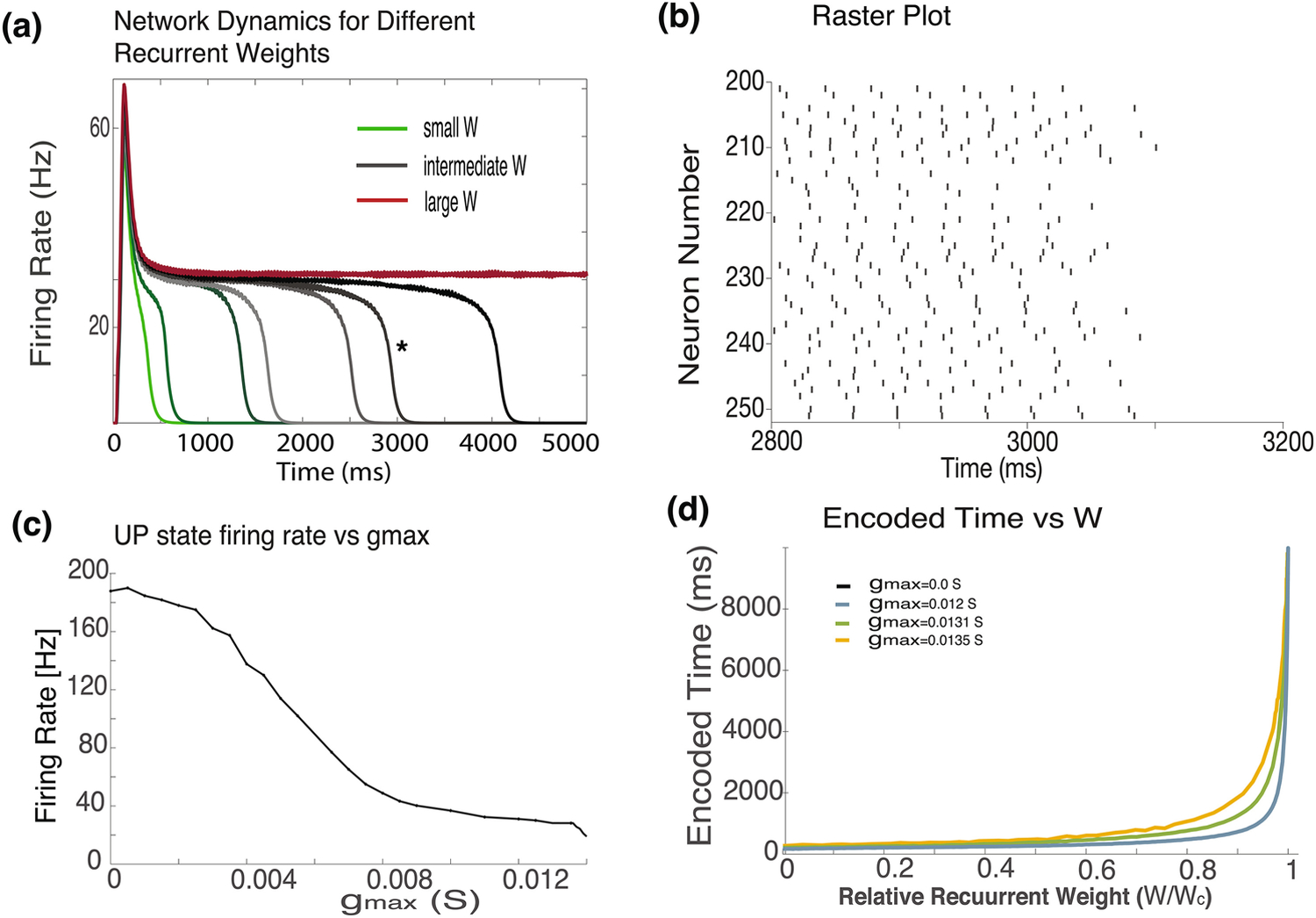
Network behavior with AIF neurons. Dynamics of recurrent networks with AIF neurons with different recurrent weights (W). For small weights (green) network activity decays rapidly, as weights increase (gray) the network dynamics exhibits a longer plateau, and at sufficiently large W the network becomes bistable. Firing rates at the plateu are moderate (30–40Hz). **(b)** Raster plot of network that decays at ~3100 ms (indicated by * in 3a). Spikes are shown for a set of 50 neurons and from 2800–3200 ms. Spike times are irregular and uncorrelated across neurons. Mean firing rate of the network when the new hybrid model is implemented. The network activity peaks around 50 Hz and drops to a plateau level at about 30Hz that maintains for extended time periods. **(c)** The relation between the firing rate just above criticality of the UP state in a bi-stable network and the conductance of the I_CAN_ channel (g_max_). As g_max_, increases the firing rate just above criticality decreases. **(d)** The T vs W curves for AIF networks with different g_max_ values. In the LIF network (g_max_ = 0, black) a very steep curve is obtained. As g_max_ is increased, the curves become progressively less steep. The recurrent weights in the x axis are normalized in that they are divided by Wc; the minimal W at which the network becomes bistable

Simulations of recurrent networks of AIF neurons with different recurrent weights are seen in figure 3a. The plateaus are maintained for extended time periods, much longer than those of the LIF networks, even though the synaptic time constant is set to 20ms. Note that with this network we are able to obtain much larger decay times than we can for the LIF model, and that the firing rates are much lower and comparable to those recorded experimentally. In the LIF network, even in the absence of additional noise, we were unable to obtain decay times larger than ~ 2400ms (Fig. 1a), with the AIF network we can obtain a decay time of over 20,000 ms. For sufficiently large recurrent efficacies, the network becomes bi-stable, that is the plateaus are maintained indefinitely, but still with moderate firing rates.

For every set of single-neuron parameters, the critical value of recurrent weights at which bi-stability is obtained is different. The firing rates of the bi-stable network just above criticality depend on g_max_, the maximal conductance of the I_CAN_ channels. When g_max_ is set to zero, the impact of the I_CAN_ currents are eliminated and the network behaves like the LIF model. As g_max_ is increased, the minimal UP state firing rate of the bi-stable network decreases monotonically (Fig 3c), reaching values that are lower than 40 Hz, for larger values of g_max_. These values are consistent with stable firing rates observed experimentally (Fuster & Alexander, 1971; Goldman-Rakic, 1995; Shuler & Bear, 2006) and are lower than rates obtained with LIF neurons even when long synaptic time constants are used (Fig. 1a). Note, that for all values of g_max_ used here, the single cell is not bi-stable.

To understand the AIF-network’s ability to represent larger decay times, we analyzed the relationship between the recurrent normalized weights and the network decay time, for AIF networks with different values of g_max_ (Fig. 3d). The weights are normalized to the critical value of weights at which the network becomes bi-stable. When g_max_ = 0, which is identical to the LIF model (Fig 1d), a steep T vs. W curve is obtained (Fig. 3d, black line). The steep curve implies that relatively small changes in W lead to very large changes in T. As g_max_ is increased the curves become progressively less steep, and small changes in W result in more moderate changes, and therefore the network is able to represent larger decay intervals.

**Figure 4:**
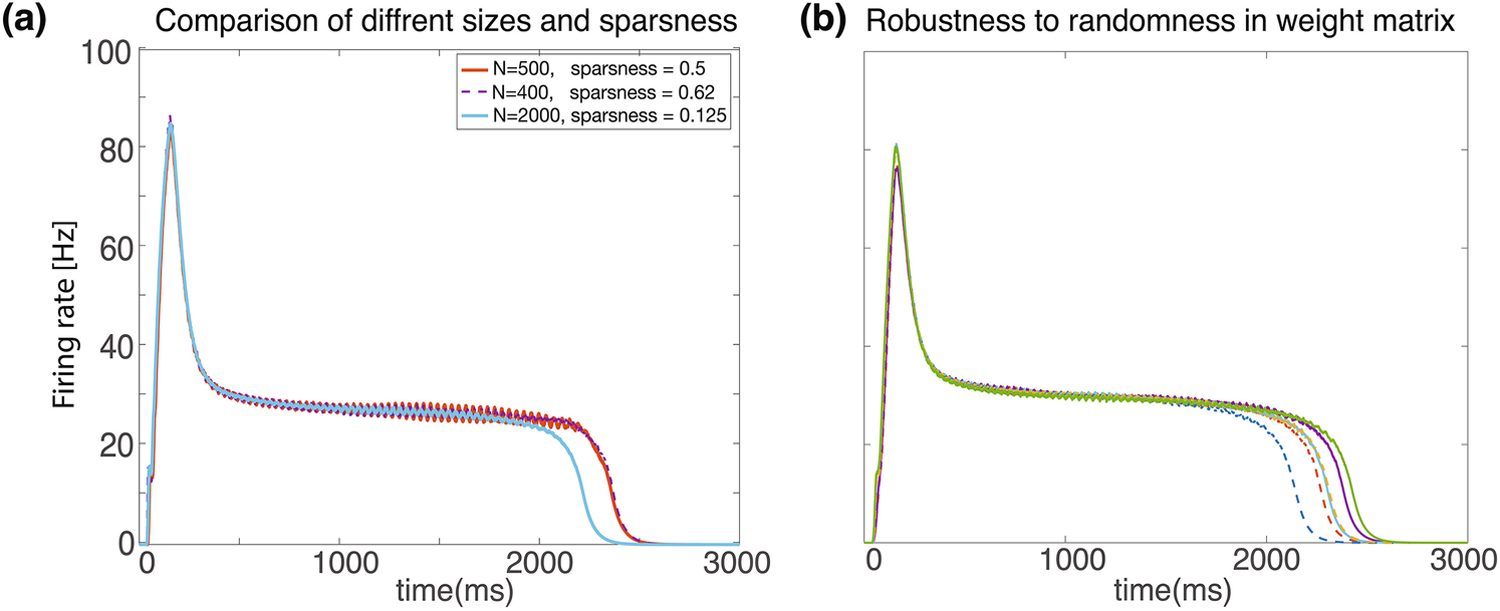
Robustness. Network dynamics are nearly identical for different sizes. Several examples shown over a range of different network sizes (N=400–2000), and levels of sparseness (0.125–0.62). Different combinations are color coded as shown in legend. **(b)** Network is robust to randomness in weight matrix. Three runs are shown for a weight matrix in which all non-zero synaptic efficacies have the same value (solid lines) and three runs in which the non-zero weights were chosen from a uniform random distribution (dashed lines). The results are quite similar

This framework described here is qualitatively robust to many variations in the scheme. As shown in the examples in figure 4a networks with many different sizes and degrees of sparsity can exhibit the similar dynamics given and appropriate synaptic efficacy parameter. Additionally, weight matrixes do not have to have identical non-zero elements. In figure 4b we compare networks in which all non-zero elements have the same value (solid lines) to networks in which weights were chosen from a uniform distribution with the same mean but a large variance (dashed lines). If we define the non-zero weights in the uniform sparse network as w_uni_, then in the randomized network the weights are chosen from a uniform distribution with the range [0 2w_uni_]. Network dynamics slightly differ from run to run due to noisy input spike trains, and random instantiation of the sparse connectivity matrixes. Network dynamics with identical non-zero weights and random weights were similar.

## 3. Learning temporal dynamics

We have shown that recurrent networks with AIF neurons significantly outperform LIF networks in terms of the range of temporal intervals they can represent. Here we show, using a previously described learning rule (Gavornik et al., 2009; Shouval & Gavornik, 2011) that AIF neurons can learn to represent these temporal intervals from stimuli paired with a delayed reward. The learning rule is based on the idea of reward dependent expression (RDE) of synaptic plasticity. The RDE rule works by generating Hebbian temporal traces, that are converted into changes in synaptic efficacies only when a reward is provided. These traces solve the temporal credit assignment over a range of seconds. The rule also stops changing efficacies once the target learning is achieved (Gavornik et al., 2009; Huertas et al., 2015; Shouval & Gavornik, 2011). When learning is complete, the network is expected to predict the timing of expected reward. We have defined learning as successful when the predictedtime is within a 15% range of the target time (|P_err_ | < 15%). The prediction error, P_err_ is defined as: P_err_ = < 100 * (E_p_/T_rew_) >; where E_p_ is the difference between the network decay time T, and the reward time T_rew_, and the < > denote running average over a set number of trials.

**Figure 5:**
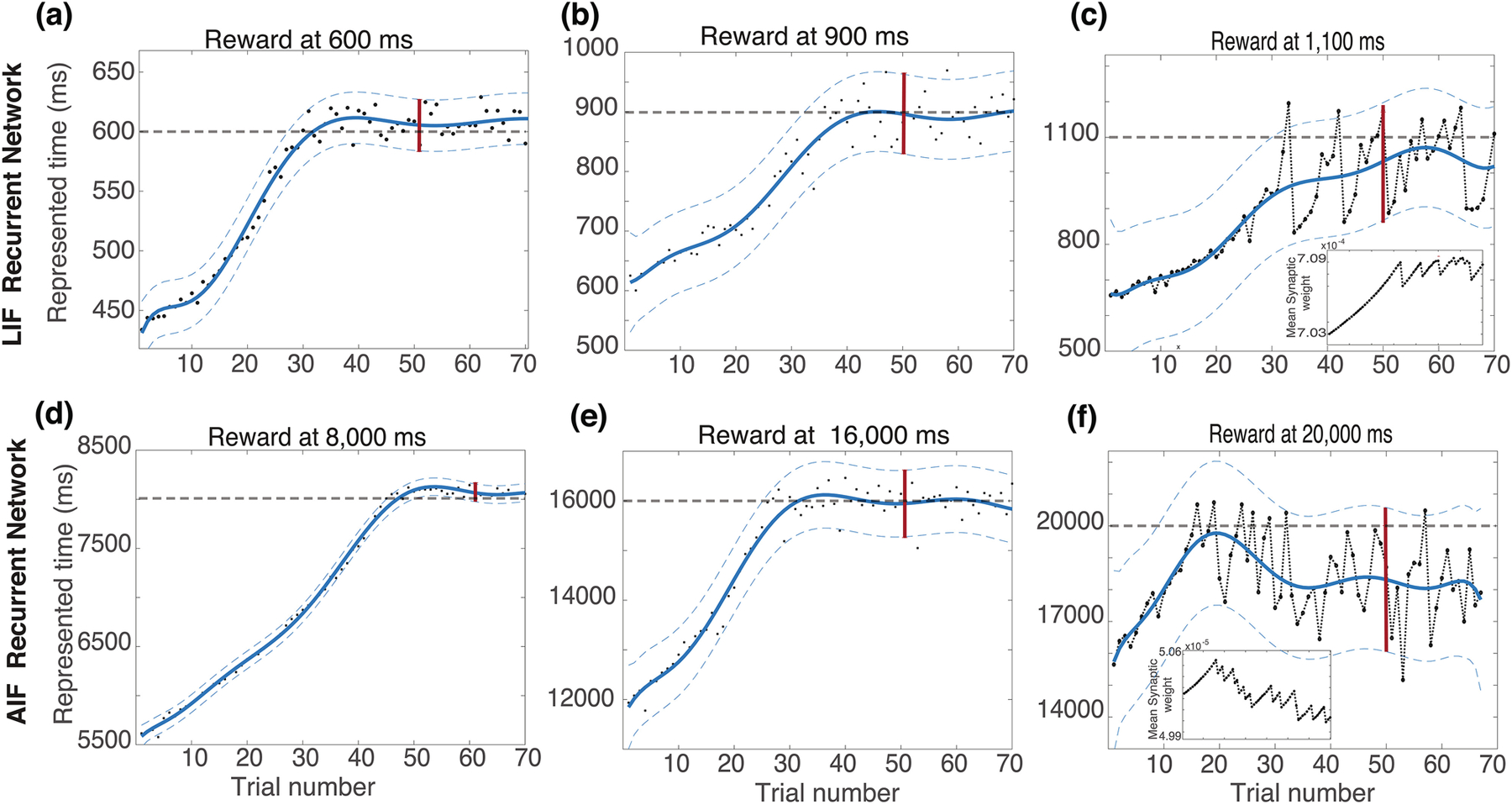
Learning reward times using RDE in recurrent networks with and without active conductances. Network decay time (T) changes are plotted with respect to training trials for different target times and for both LIF (top plots) and AIF (bottom plots) models. Learning reward times with LIF model (top). For 600ms, and 900ms **(a** and **b)** learning is stable, but for 1100ms (**c**), the learning leads to large fluctuations. Inset shows weight fluctuations which lead to large decay time fluctuations. Learning reward time AIF model (bottom). For 8,000ms, and 16,000ms **(d** and **e)** but for 20000ms **(f),** learning leads to large fluctuations. In all plots, thick dashed gray lines show target decay times, filled circles decay time on single trial, blue line is a moving average, thin dashed lines confidence interval of the decay times, and vertical red bar is the confidence interval used for determining if learning is stable

We have compared the ability to train LIF and AIF networks with RDE over a large range of target times (Fig. 5). Each subplot of figure 5 shows the network decay time T, as a function of the training trial number. On the top (Fig. 5a−c) this is shown for the LIF model, and on the bottom (Fig. 5d−f) for the AIF model. Initial weights at each subplot were not zero, and therefore the initial T, is not zero either. During training, the duration of the network activity increases for each trial until the reward time is reached and stabilizes close the target. The fluctuations around the target reward line are used to calculate P_err_ (red bar). If the fluctuations are high in when divided by target delay period (|P_err_| > 15%, the training is deemed unsuccessful.

Training for target times of 600 and 900ms using the LIF model is successful (Fig. 5a,b). As the delay is increased from 600 to 900ms, the fluctuations increase from 40ms to 150ms, giving 6.7% and 15% prediction error (P_err_), respectively. The network is unable to sufficiently stabilize its synaptic efficacy values and dynamics when attempting to learn longer delays. For a target decay time of 1100ms, we obtain P_err_ = 40%, significantly above the target fluctuation of 15% that we have defined as our threshold for successful training. In contrast we were able to successfully train the AIF for up to 16,000ms delayed reward (Fig. 5d,e). For 8,000ms and 16,000ms target decay times, the prediction errors were 2.5% and 9.4% P_err_, respectively (Fig. 5d,e). For a 20,000ms target, the network had a prediction error, P_err_ of 19%, slightly above our target cutoff error.

Apart from the ability to represent much longer temporal intervals, recurrent AIF networks also exhibit firing rate dynamics, and specifically firing rates that are more consistent with experimental results.

**Figure 6.**
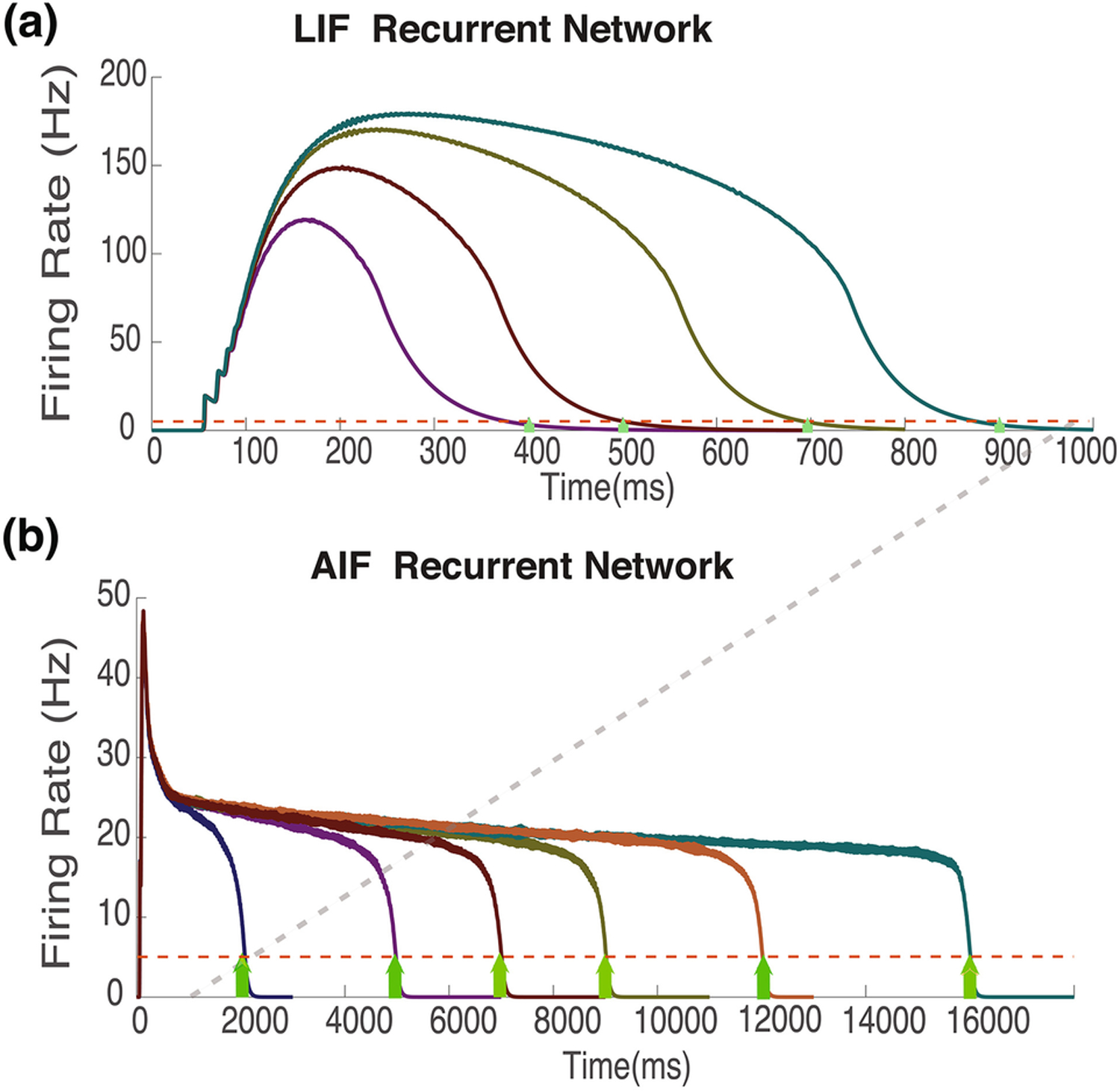
Temporal dynamics in trained recurrent networks with LIF and AIF Neurons. Average network firing-rate activity for trained networks using LIF neurons **(a)** and AIF neurons **(b).** Yellow arrows represent target training times, and dashed red line represents target firing rate at target time. Note the different scales of the x and y axes. The dashed gray line illustrates the different x axis scale

In figure 6, we compare the temporal firing rate patterns for the LIF model (top) and the AIF model (bottom) which are trained to different target reward times, shown with green arrows. Trained LIF networks result in unreasonably high firing rates. For longer duration targets, these exceed 100Hz (Fig. 6a); rates that are not characteristic of experimentally observed results. In contrast, the levels of the transient plateaus for the AIF model are between 20–30Hz (Fig 6b) for every reward delay for which training was successful. Such rates are consistent with experimental results (Namboodiri et al., 2015; Shuler & Bear, 2006).

## 4. Methods

### 4.1 The Network structures

The goal of this study is to examine the impact of intrinsic conductances, here high voltage activated calcium channels, on network dynamics and synaptic plasticity. We first analyze the behavior of the network built up with basic LIF neurons, investigate the capacity of the model for learning and looking at the network’s response for a delayed reward task. Later, we implement the g^can^ conductance (Eq. 11) to represent the AIF neuron model and perform the same analysis keeping the previous parameters identical. Same network structure is preserved for both models to have a solid comparison.

Our goal here is to specifically elucidate the role of intrinsic conductances, we have therefore chosen the simplest network form in order to reduced unnecessary complexity. The network is composed of randomly and sparsely connected, (N = 1000), excitatory neurons, with a sparsity of 10%. The network gets activated through a transient feedforward Poisson input “ I^ext^” (Eq. 2) initiated from an outside population of 1000 neurons. The connections among those two populations are sparse with an all-to-all 10% connectivity. The membrane potential of each post synaptic neuron, i, is described by conductance based leaky-integrate and fire model. In the absence of the external input, the activity of the network is maintained through the recurrent connections. The duration of the delay period is correlated with the summation of the synaptic transmission and intrinsic conductance if activated.

We also tested robustness to this connectivity scheme. In figure 4a we changed the network size between N = 400 to N = 2000, and varied the sparsity between 12.5% to 50%. In figure 4b we chose non zero weight matrix elements from a uniform distribution, with the same mean of the networks with the fixed non-zero matrix elements. If the non-zero weight matrix elements of the non-random matrix had a value w_uni_, then in the randomized matrixes we chose values in the range [0 2w_uni_], which have the same mean.

The details of the basic LIF based model and AIF model networks are explained in the following sections.

### 4.2 LIF neurons and recurrent network

The membrane voltage of a single neuron is constructed by (a) the leakage term, (b) the excitatory feedback current and (c) feedforward input; I^leak^, I^rec^ and, I^ext^, respectively. (Eq. 1) As the membrane potential reaches the threshold level, the neuron fires an action potential.


(1) $${\text{C}}_{\text{m}}\frac{\text{d}}{\text{dt}}{\text{V}}_{\text{i}}\left(\text{t}\right)={\text{I}}_{\text{i}}^{\text{leak}}\left(\text{t}\right)+{\text{I}}_{\text{ij}}^{\text{rec}}\left(\text{t}\right)+{\text{I}}_{\text{ij}}^{\text{ext}}\left(\text{t}\right)$$

(a) The leakage term I^leak^, represents the role of the summed ion channels and pumps dragging the voltage down to resting membrane potential, E^I^. (Eq. 2)

(2) $${\text{I}}_{\text{i}}^{\text{leak}}=-{\text{g}}^{\text{leak}}\left({\text{V}}_{\text{i}}\left(\text{t}\right)-{\text{E}}_{\text{i}}^{\text{I}}\right)$$

(b) I^ext^” is the input received from the external population (Eq. 3). The input conductance “ g^ext^” is dynamic, modulated by instant synaptic activity levels, ${\text{S}}_{\text{i}}^{\text{ext}}$, and the synaptic strengths “${\text{J}}_{\text{i}}^{\text{ext}}\left(\text{t}\right)$”of each input node. (Eq. 4) At each time step, synaptic transmission “${\text{S}}_{\text{i}}^{\text{ext}}$”, is updated at the post synaptic neuron, for the active nodes. Each presynaptic spike adds to the synaptic activity by “ρ_s_” of the available post synaptic receptors, $\left(1-{\text{S}}_{\text{i}}^{\text{ext}}\left(\text{t}\right)\right)$. (Eq. 5)

(3) $${\text{I}}_{\text{i}}^{\text{ext}}=-{\text{g}}_{\text{i}}^{\text{ext}}\left({\text{V}}_{\text{i}}\left(\text{t}\right)-{\text{E}}_{\text{i}}^{\text{ext}}\right)$$

(4) $${\text{g}}_{\text{i}}^{\text{ext}}={\text{S}}_{\text{i}}^{\text{ext}}\left(\text{t}\right){\text{J}}_{\text{i}}^{\text{ext}}\left(\text{t}\right)$$

(5) $$\frac{\text{d}}{\text{dt}}{\text{S}}_{\text{i}}^{\text{ext}}\left(\text{t}\right)=-\frac{1}{{\text{τ}}_{\text{s}}}{\text{S}}_{\text{i}}^{\text{ext}}\left(\text{t}\right)+{\text{ρ}}_{\text{s}}\left(1-{\text{S}}_{\text{i}}^{\text{ext}}\left(\text{t}\right)\right)\sum_{\text{j}}^{\text{n}}\text{δ}\left(\text{t}-{\text{t}}_{\text{j}}\right)$$

(c) Each neuron receives an excitatory feedback current, I^rec^ (Eq. 6), from approximately 10% of the recurrently connected network. As the presynapticneuron fires an action potential at time t_j_, the conductance of the post synaptic neuron, ${\text{g}}_{\text{i}}^{\text{rec}}$ (Eq. 8), is enhanced as a consequence of activated synaptic transmission, “S^rec^” (Eq. 7).


(6) $${\text{I}}_{\text{i}}^{\text{rec}}=-{\text{g}}_{\text{i}}^{\text{rec}}\left({\text{V}}_{\text{i}}\left(\text{t}\right)-{\text{E}}_{\text{i}}^{\text{rec}}\right)$$

(7) $$\frac{\text{d}}{\text{dt}}{\text{S}}_{\text{i}}^{\text{rec}}\left(\text{t}\right)=-\frac{1}{{\text{τ}}_{\text{s}}}{\text{S}}_{\text{i}}^{\text{rec}}\left(\text{t}\right)+{\text{ρ}}_{\text{s}}\left(1-{\text{S}}_{\text{i}}^{\text{rec}}\left(\text{t}\right)\right)\sum_{\text{j}}^{\text{n}}\text{δ}\left(\text{t}-{\text{t}}_{\text{j}}\right)$$

(8) $${\text{g}}_{\text{i}}^{\text{rec}}={\text{S}}_{\text{i}}^{\text{rec}}\left(\text{t}\right){\text{J}}_{\text{i}}^{\text{rec}}\left(\text{t}\right)$$

We use different time constant of recurrent connections from 20–100 ms. In the slow end of this range synaptic conductance has a value similar to that of NMDA receptors, however we have not incorporated here the voltage dependence of NMDA receptors. Note also that these synaptic efficacies saturate at higher presynapticfiring rates due to the $\left(1-{\text{S}}_{\text{i}}^{\text{rec}}\left(\text{t}\right)\right)$ in the dynamical equations of synaptic efficacy. Addition of the limiting term is methodologically sound because there is a maximal level of receptors and bound receptors cannot be bound again. Saturation is often ignored for receptors with fast time constants because at moderate firing rates fast receptors are far from saturation. Since we vary our receptor time constants over a large range, and since we simulate networks that attain high firing-rates which are not experimentally realistic we found it simpler to include saturation in all of our synaptic conductances.

### 4.3 AIF neuron model

For the AIF neuron model, in addition to the leakage term, feedforward and feedback input, each neuron is implemented with both calcium dependent non-selective cation current, I^can^, and high voltage activated (HVA) calcium channels. (Eq. 9) (Fig. 2a)

(9) $${\text{C}}_{\text{m}}\frac{\text{d}}{\text{dt}}{\text{V}}_{\text{i}}\left(\text{t}\right)={\text{I}}_{\text{i}}^{\text{leak}}\left(\text{t}\right)+{\text{I}}_{\text{ij}}^{\text{rec}}\left(\text{t}\right)+{\text{I}}_{\text{i}}^{\text{can}}\left(\text{t}\right)+{\text{I}}_{\text{ij}}^{\text{ext}}\left(\text{t}\right)$$

The HVA calcium conductance is active for depolarized membrane voltages of −20mV and higher(Lacinova, 2005). This condition is met only during the fast action potential window since the threshold for generating an ction potential is set to −55mV. With each action potential, ρ amount of calcium fuses into the cell, and the intracellular calcium concentration is calculated by the Equation 10.


(10) $$\frac{\text{d}}{\text{dt}}\left[\text{Ca}\right]\left(\text{t}\right)=-\frac{\left[\text{Ca}\right]}{\text{τCa}}+\text{ρCa}\sum_{\text{j}}^{\text{n}}\text{δ}\left(\text{t}-{\text{t}}_{\text{j}}\right)$$

The intracellular calcium concentration level, [Ca], modulates the dynamics of the non-selective cation conductance, “g^can^ ”. As seen in figure 2b, g^can^ gets activated during the transient input window, reaches to its maximum value and stays open until the calcium concentration gets low. The g^can^ conductance is represented by a hill function (Eq. 11), where g_max_ is the maximum conductance limit the I^can^ channels can hold (Eq. 12).


(11) $${\text{g}}_{\text{i}}^{\text{can}}\left(\left[\text{Ca}\right]\right)={\text{g}}_{\text{max}}\frac{{\left[\text{Ca}\right]}^{\text{m}}}{{\left[\text{Ca}\right]}^{\text{m}}+{\text{θ}}_{\text{Ca}}^{\text{m}}}$$

(12) $${\text{I}}_{\text{i}}^{\text{can}}\left(\text{t}\right)=-{\text{g}}_{\text{i}}^{\text{can}}\left({\text{V}}_{\text{i}}\left(\text{t}\right)-{\text{E}}_{\text{i}}^{\text{can}}\right)$$

The addition of the I^can^ currents creates an intracellular feedback mechanism where the activity of the cell activates the I^can^ conductance and the I^can^ currents enhances the cellular activity in return.

### 4.4 The learning rule

The plasticity rule used here is the reward dependent expression rule (RDE) which has been shown to solve the temporal credit assignment problem (Gavornik et al., 2009; Huertas et al., 2015; Shouval & Gavornik, 2011).

In order to implement this rule, a moving temporal average of the firing rate for neuron “i” is calculated by: ${\text{τ}}_{\text{r}}\frac{\text{d}}{\text{dt}}{\text{R}}_{\text{i}}\left(\text{t}\right)=-{\text{R}}_{\text{i}}\left(\text{t}\right)+\sum_{\text{k}}\text{δ}\left(\text{t}-{\text{t}}_{\text{i},\text{k}}\right)$, where τ_r_ is the width of the exponential time window, and t_i,k_ are the times of the k^th^ spike in the i^th^ neuron.

Using this variable, a Hebbian is calculated for each recurrent synapse between neuron “i” and “j” such that:

(13) $${\text{H}}_{\text{ij}}\left(\text{t}\right)={\text{R}}_{\text{i}}\left(\text{t}\right){\text{R}}_{\text{j}}\left(\text{t}\right)$$

In order to implement RDE we calculate synaptic eligibility traces: ${\text{L}}_{\text{ij}}^{\text{p}}\left(\text{t}\right)$ by the equation:

(14) $${\text{τ}}_{\text{p}}\frac{\text{d}}{\text{dt}}{\text{L}}_{\text{ij}}^{\text{p}}\left(\text{t}\right)=-{\text{L}}_{\text{ij}}^{\text{p}}\left(\text{t}\right)+{\text{H}}_{\text{ij}}\left(\text{t}\right)$$

These eligibility traces are only converted to long lasting synaptic efficacies, “L_ij_” (Eq. 15), when a reward (r_0_) is delivered. The value of r_0_ is the target activity level at time of reward, and in order to stop learning when this value is attained the effective reward used is:

(r_0_(t) − R_i_(t)), where R_i_(t) is the firing rate of the i’th neuron.


(15) $$\frac{\text{d}}{\text{dt}}{\text{L}}_{\text{ij}}\left(\text{t}\right)=\text{η}{\text{L}}_{\text{ij}}^{\text{p}}\left(\text{t}\right)+\left({\text{r}}_{0}\left(\text{t}\right)-{\text{R}}_{\text{i}}\left(\text{t}\right)\right)$$

### 4.5 Parameters

S = 10%

N = 1000

Cm = 1μF

$${\text{E}}_{\text{i}}^{\text{I}}=0\text{mV}$$

$${\text{E}}_{\text{i}}^{\text{ext}}=55\text{mV}$$

$${\text{J}}_{\text{i}}^{\text{ext}}=0.021\text{mS}$$

τ_s_ = 20ms

ρ_s_ = 1/7

$${\text{E}}_{\text{i}}^{\text{rec}}=55\text{mV}$$

$${\text{J}}_{\text{i}}^{\text{rec}}=0\text{mV}$$

τ_Ca_ = 100ms

ρ_Ca_ = 0.0787

G^can^ = 0.0135 S

$${\text{θ}}_{\text{Ca}}^{\text{m}}=1$$

m = 4

$${\text{E}}_{\text{i}}^{\text{can}}=80\text{mV}$$

τ_r_ = 50ms

τ_p_ = 5000ms

r_0_ = 4.5Hz

η = 10^−6^ to 10^−9^

In the simulations in figure 4, in which we tested robustness, we changed these parameters, and these specific changes are indicated in the text and the figures.

## 5. Discussion

Single neurons are highly complex and they possess many intrinsic active conductances that contribute significantly to the function of neural circuits. In contrast, many theoretical circuit models ignore single neuron complexity and use instead highly simplified models of the single neurons. This simplified approach is justified because it helps understand the role of the circuit itself, but it might not faithfully represent the properties of a circuit composed of more complex neurons. Generally, intrinsic properties of single neurons can and do affect circuit dynamics (Jin et al., 2007; Marder et al., 1996). In this paper we demonstrate how a specific set of intrinsic conductances can affect the dynamics of bi-stable and slowly decaying networks.

Recurrent networks can exhibit bi-stability, in which the network activity can be either in a low or high activity state which lasts indefinitely (Compte et al., 2000). Networks with the same type of structure, but at parameters that are subcritical for bi-stability can exhibit slow transient dynamics(Gavornik et al., 2009). For both of these cases slow synaptic dynamics, on the order of 100ms are typically assumed for the networks to quantitively approach physiological measurements of firing rates and possible decay times, and in some systems such long time-constants might be justified (Wang et al., 2013). In this paper, we examined if the addition of specific active conductances to the single neuron model can improve the circuit behavior, in the absence of slow synaptic conductances. We chose a combination of I_CAN_ and voltage gated calcium channels that form a subthreshold positive feedback loop, which acts as a conditional slow intrinsic time constant. We show that by including these channels, we improve significantly the agreement between the network performance and experimental results. With active intrinsic conductances, the bi-stable network achieves bi-stability at much lower firing rates than obtained by a network with fast conductances, and even lower than the networks with NMDA-like slow synaptic time constants. These results are in much better agreement with firing rates observed experimentally (Fuster and Alexander, 1971; Goldman-Rakic, 1995). We have also shown that the slowly decaying networks have plateaus at much lower firing rates, consistent with experimental results (Namboodiri et al., 2015; Shuler & Bear, 2006). In this subthreshold mode, the network can realistically exhibit decays of up to 16 seconds, much larger than can be accomplished with networks of IAF neurons with fast or even slow synaptic time constants alone. This network can also be trained, with a biophysically plausible learning rule, to decay at short or long intervals over a much larger range than networks with relatively slow synaptic time constants (Gavornik & Shouval, 2011; Gavornik et al., 2009). We have also shown that these networks with AIF neurons are robust to network size, degree of sparseness, and randomness in the recurrent connectivity matrix. Moreover, they exhibit biologically plausible spike rasters.

The single cell mechanisms assumed here are inspired by previous experimental papers that observed persistent activity in single cells in various brain regions (Egorov et al., 2002; Fransén et al., 2006; O’Malley et al., 2020; Rahman & Berger, 2011) and by the dependance of this persistent activity on non-specific cationic channels and calcium currents, as identified in those papers. This work is also based on previous single cell models of such observations (Egorov et al., 2002; Fransén et al., 2006; Shouval & Gavornik, 2011). However, other experiments in slices (Winograd et al., 2008) and cultures (Volman et al., 2007) have indicated alternative mechanisms that can lead to slow time constants and to persistent or reverberating synaptic plasticity. It is quite feasible that such alternative mechanisms that generate effective slow time constants in single neurons or single synapses would produce qualitatively similar results to those described here. Indeed, it is quite likely that any mechanism that generates a conditional slow time constant in single neurons or synapses will have a similar effect on circuit dynamics. Such a mechanism is conditional in the sense that the slow time constant are turned on only when cellular activity exceeds a threshold, such that onset dynamics are still rapid, but the decay dynamics are slowed down.

revious work (Tegnér et al., 2002) has simulated recurrent networks with using more realistic and complex single cell models, and in that case as well a large NMDA/AMPA ratio is typically required. However, this paper also explored a similar mechanism to the one proposed here, in which I_CAN_ channels were added to the single neurons which also had voltage gated calcium channels. The Tengér et al. (2002) paper has shown that the addition of I_CAN_ channels lowers the minimal NMDA/AMPA ratio that is required for attaining bi-stability. However, this previous publication did not explicitly investigate how such intrinsic active condutances affect the firing rates in the active state, it did examine how it affects the spike statistics of the slowly decaying network, how it extends the range of decay times of a slowly decaying networks by an order of magnitude or how it enables a learning rule based on reward dependent synaptic plasticity (Gavornik et al., 2009) to learn decay times of up to 16 seconds.

In order to obtain bi-stability with realistic firing rates in the UP state, simply adding a recurrently connected inhibitory population is not a solution. Adding a population of recurrent inhibitory neurons without changing other parameters will indeed reduce firing rates, but it will also destabilize the UP state. In order to restabilize the UP state excitatory conductances can be increased resulting in an increase in firing rates. Networks that receive external input, even with fast intrinsic time constants can exhibit bi-stability with lower firing rates in the UP state (Renart et al., 2006; Shouval & Gavornik, 2011). Such networks are not self-sustained, since attaining bi-stability depends on this external input (Renart et al., 2004, 2006). When such networks include balanced excitatory and inhibitory conductances they can also attain bi-stability in which spike count variability is high in both the UP and DOWN states, consistent with experimental observations (Renart et al., 2006). This fluctuation driven bi-stability requires fine tuning of the ratio between excitatory and inhibitory weights. In addition, networks that can sustain an UP state with experimentally realistic firing rates, due to an external current still have very steep T vs W curves, similar to those in figure 1c. Therefore, it is not simple to use such a model in combination with synaptic plasticity of excitatory weights, which alone will easily move the network out of the balanced, fluctuation driven state, resulting in high firing rates, and low variability. Moreover, such networks could not be trained to generate long-duration transients that are longer than those that can be learned by a self-sustaining network of LIF neurons with fast conductances.

Another use of recurrent networks is to produce integrator-like networks. Such networks have a continuum of fixed points and the activity level at each fixed point is proportional to the integral of an external signal. At the fixed points of such networks, the leak term is exactly equal to the feedback term that results from the recurrent network. The fixed points of such integrator networks are highly sensitive to their parameters, and very small variability in such parameters can result either in a decay or an explosion in network activity. Several approaches to overcome such ultra-sensitivity of been proposed (Goldman et al., 2003; Koulakov et al., 2002). Robustness in these models arises from the networks being composed of robust hysteretic sub-networks (Koulakov et al., 2002), or the existence of hysteretic subunits in dendrites (Goldman et al., 2003). Interestingly the hysteretic sub-networks have also been assumed to required NMDAR like receptors, either for their slow dynamics, or because of the voltage dependence of the NMDAR receptors (Koulakov et al., 2002). Similarly, the hysteretic dendritic compartments are also assumed to have slow time constants which are assumed to arise from slow calcium channels or NMDA receptors (Goldman et al., 2003). Models of sensory integration or of decision making also employ recurrent networks. Such models might be multi-stable and the different states represent decisions or sensory processing. In such models, activity in the network depends on a persistent external input, and they do not maintain the firing of the network solely due to feedback in the recurrent network, and therefore do not need to maintain as high a firing rate while persistently active. However, in practice such models also typically assume that excitatory recurrent connections are dominated by slow, NMDAR-like, synaptic transmission (Wang, 2002; Wimmer et al., 2015).

Although we have added some biologically realistic complexity to our neural model, real neurons in the brain are much more complex, they include active sodium and potassium conductances that are necessary for spiking and a slew of other active conductances, which are differentially expressed in different types of neurons. Neurons also have a complex spatial structure with different types of compartments that also express different channel types. The neuron used here is still very simple, it is a single compartment model with only two additional channels expressed. Action potentials, in the model, are still simply generated by threshold crossing. Obviously, such a simple model is also not a faithful representation of real cortical neurons. We adopt the approach in order to understand what role such channels can play, and demonstrate that with such channels, firing statistics in networks have more realistic properties and the networks are more robust. By using this conservative approach for adding complexity, we can interpret the model and understand the possible role of such channels, at the possible cost of reduced biological realism. The networks used here are simplified in other respects as well, for example they do not include any inhibitory neurons. Although these recurrent networks either with LIF or AIF neurons, are composed of only excitatory neurons the simple addition of an unstructured, randomly connected, population of inhibitory neurons does not qualitatively change the network behavior. An addition of recurrently connected inhibitory neurons, without any other parameter changes, will clearly reduce the firing rates of the network, destabilize bi-stability and eliminate the slow decay. However, an increase of the recurrent excitatory efficacies can reestablish both these behaviors, without significant qualitative differences in firing rates in the UP state, or the shapes of the decay times vs. recurrent weight curves. In contrast, an addition of structured inhibitory connections can have a more profound effect on network dynamics. Structured connections can for example be used to generate competitive networks that can be used for decision making (Wang, 2002; Wimmer et al., 2015), or to generate different classes of neuronal dynamics within the network (Huertas et al., 2015). The analysis of such network dynamics is beyond the scope of the current paper.
